# Spacetime

**DOI:** 10.5935/abc.20160022

**Published:** 2016-02

**Authors:** Aloyzio Achutti

**Affiliations:** Programa de Pesquisa e Extensão sobre Saúde Urbana, Ambiente e Desigualdade (UFRGS), Porto Alegre, RS - Brazil

**Keywords:** Cardiovascular Diseases/mortality, Disease Prevention, Social Inequity, Demographic Data

Spacetime is a fundamental issue in physics, inherent to the basis of science. It encompasses two concepts bound to the perception of our simple reality, through which we measure and interpret our universe.

The notion of space relates to sovereignty, a characteristic that matters to all living beings to ensure species subsistence, reproduction, safety and preservation. Human beings have evolved to urban organization, a way of living together that has predominated for a few decades.

Time is embedded in DNA itself, through the phenomenon of apoptosis, which controls each actor's time on stage, ensuring permanent renovation and elimination of mistakes made along the way - a strategy to guarantee that the show goes on - prioritizing the species over the individual. It relates to mortality, evolution, development and sustainability.

Mortality describes the mean lifespan of a population, yielding the illusion of something homogeneous, but clearly unequal regarding its distribution, including the spatial one. The search for the cause of that inequality leads us to speculate about both the life quality and conditions of the population and the heterogeneity in human development. The comparison of historical series allows us to identify marginalized strata, or those unable to take advantage of something others benefit from.

That type of study comparing historical series can be carried out on a world scale, observing, analyzing and comparing one country with another. Or it can be carried out inside one country with several distributions of the spatial matrix, according to regions, states or municipalities. In addition, the study can be conducted according to gender, age groups, ethnicity, occupations, etc.

The study of mortality evolution over time in the Rio de Janeiro state^1^ is a good example. We, in the city of Porto Alegre, worried with the urban issue, have studied our city and its distribution according to its districts.^2^ That study has evidenced, through the early mortality due to cardiovascular diseases, the vulnerability related to social inequality and urban segregation. One of the authors, Sérgio Bassanesi, used to say that as you move away from the center to the periphery of a city (from better to worse indices), more years of potential life are lost. Or as I have discussed on a newspaper article at the time,^3^ "tell me your zip code, and I will tell you your cardiovascular risk".

Historical series allow indicators to be put into perspective. That study on mortality in the Rio de Janeiro state has shown a progressive and consistent drop in mortality rates over time in almost all regions assessed, most of them converging to better and relatively closer levels, despite the persistence of a delay among individuals coming from less favorable situations, which would be expected.

Our trend towards professional formation (or deformation) is to focus on the good news related to health indicators, forgetting that they are no more than an epiphenomenon, determined by quality of life, and that not always a reduction in mortality reflects better performance of the other human development indicators. Not only health, or more specifically cardiovascular health, is linked to social determinants, but violence is linked to inequality, to self-esteem defects, to apathy and lack of perspective, to savage work exploitation, to financial and budget imbalances, to corruption, to quality of care and information, to all wrongdoing that affects people, in Brazil or anywhere else. To keep advantages and for protection, we divide ourselves territorially (space), as if we were not occupying the same space at the same time, and as if we did not belong to the same species.

We have been worried about global warming, estimating how much time we still have to prevent a global catastrophe, avoiding the point of no return. We should do the same when analyzing the temperature of inequality in the studies on mortality. How much time do we still have to understand the causes of inequality in the spatial distribution of health indicators? How much time is left to invest in eliminating them?
